# Bacterial artificial chromosome derived simian varicella virus is pathogenic *in vivo*

**DOI:** 10.1186/1743-422X-10-278

**Published:** 2013-09-08

**Authors:** Christine Meyer, Jesse Dewane, Kristen Haberthur, Flora Engelmann, Nicole Arnold, Wayne Gray, Ilhem Messaoudi

**Affiliations:** 1 Vaccine and Gene Therapy Institute, Oregon National Primate Research Center, Beaverton, OR 97006, USA; 2 Molecular Microbiology and Immunology Department, Oregon National Primate Research Center, Beaverton, OR 97006, USA; 3 Division of Pathobiology and Immunology, Oregon National Primate Research Center, Beaverton, OR 97006, USA; 4 Division of Biomedical Sciences, University of California-Riverside, Riverside, CA 92508, USA; 5 Department of Microbiology and Immunology, University of Arkansas, Little Rock, AK 72205, USA; 6 School of Medicine, University of California-Riverside, 900 University Avenue, Riverside, CA 92521, USA

**Keywords:** Herpesvirus, Simian varicella virus, Rhesus macaque, Bacterial artificial chromosome, Pathogenesis

## Abstract

**Background:**

Varicella zoster virus (VZV) is a neurotropic alphaherpesvirus that infects humans and results in chickenpox and herpes zoster. A number of VZV genes remain functionally uncharacterized and since VZV is an obligate human pathogen, rigorous evaluation of VZV mutants *in vivo* remains challenging. Simian varicella virus (SVV) is homologous to VZV and SVV infection of rhesus macaques (RM) closely mimics VZV infection of humans. Recently the SVV genome was cloned as a bacterial artificial chromosome (BAC) and BAC-derived SVV displayed similar replication kinetics as wild-type (WT) SVV *in vitro*.

**Methods:**

RMs were infected with BAC-derived SVV or WT SVV at 4x10^5^ PFU intrabronchially (N=8, 4 per group, sex and age matched). We collected whole blood (PBMC) and bronchoalveolar lavage (BAL) at various days post-infection (dpi) and sensory ganglia during latent infection (>84 dpi) at necropsy and compared disease progression, viral replication, immune response and the establishment of latency.

**Results:**

Viral replication kinetics and magnitude in bronchoalveolar lavage cells and whole blood as well as rash severity and duration were similar in RMs infected with SVV BAC or WT SVV. Moreover, SVV-specific B and T cell responses were comparable between BAC and WT-infected animals. Lastly, we measured viral DNA in sensory ganglia from both cohorts of infected RMs during latent infection.

**Conclusions:**

SVV BAC is as pathogenic and immunogenic as WT SVV *in vivo*. Thus, the SVV BAC genetic system combined with the rhesus macaque animal model can further our understanding of viral ORFs important for VZV pathogenesis and the development of second-generation vaccines.

## Introduction

Varicella zoster virus (VZV) is a neurotropic alphaherpesvirus and the etiological agent of varicella (chickenpox) and herpes zoster (shingles). VZV establishes latency within the sensory ganglia, and reactivation from latency can cause significant morbidity and occasionally mortality in older and immunocompromised individuals. Currently the FDA vaccine Zostavax® reduces the incidence of shingles by 51% and the burden of disease by approximately 61% [1,2]. Thus, a significant portion of vaccine recipients still remains susceptible to VZV reactivation. To improve vaccine efficacy, we need to determine the function of the viral open reading frames (ORFs) that contribute to VZV pathogenesis and those that are important for the host immune response.

Simian varicella virus (SVV) is a homolog of VZV that causes varicella-like disease and establishes latency in sensory ganglia of rhesus macaques [3-5]. SVV shares significant DNA homology and genome colinearity with VZV [6-9]. VZV and SVV have the smallest genomes of the herpesvirus family. VZV encodes at least 70 unique ORFs and SVV encodes 69 distinct ORFs [8,10]. Despite the smaller genome size and homology to herpes simplex virus (HSV), a number of VZV/SVV genes remain functionally uncharacterized. Studies characterizing viral gene function utilizing *in vitro* tissue culture models do not always model the complex host-pathogen relationship that occurs *in vivo*. Recently, with the construction of an infectious SVV bacterial artificial chromosome (BAC), the production of mutations and deletions in specific SVV ORFs will allow the investigation of gene function during *in vivo* infection [11]. Previously, SVV BAC was shown to generate infectious virus with molecular properties and *in vitro* replication kinetics comparable to wild-type (WT) SVV [11]. SVV BAC was also used to generate an ORF10 deletion virus, which demonstrated that SVV ORF10 is nonessential for replication *in vitro*[11]. In the current study we further investigate SVV BAC *in vivo* by monitoring replication kinetics, immune response and establishment of latency in rhesus macaques and show that SVV BAC is as pathogenic as WT SVV.

## Results

### Whole-genome analysis of SVV BAC

**Figure 1:**
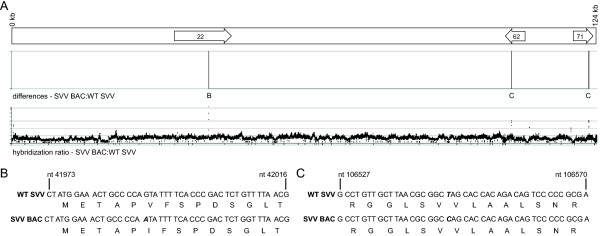
**Comparative genomic hybridization and sequence analysis comparing SVV BAC to WT SVV. A)** Schematic representation of the SVV genome highlighting the SVV ORFs (arrows) that contain sequence changes. Sequence variation results in different hybridization intensities indicated by the hybridization ratio between SVV BAC and WT SVV and signal potential nucleotide changes. **B** and **C)** The regions containing sequence variations were amplified by PCR and directly sequenced. Sequencing identified: **B)** within ORF 22 a transition occurs from G to A resulting in a missense mutation, **C)** within ORF 62/71 a transition from T to C results in a silent mutation (note the nucleotide and position number refers to the genomic position). Nucleotide substitution (bold italics).

The BAC derived SVV viral genome was comprehensively analyzed by comparative genomic hybridization (CGH) and directly compared to wild-type (WT) SVV. Using this technique, any differences in genomic sequence between SVV BAC and WT SVV results in variations in hybridization intensities to corresponding segments represented on the array, giving an altered hybridization ratio between SVV BAC and WT SVV (Figure 1A). CGH analysis revealed that two areas displayed variations when compared to WT SVV, indicating differences in nucleotide sequence at these locations. These regions were amplified via PCR and directly sequenced resulting in the identification of two nucleotide substitutions that produced 1 missense mutation and 1 silent mutation within the coding region of the SVV BAC genome. Specifically, we identified a point mutation at nucleotide 41990 from G to A within ORF22, producing an amino acid change from valine to isoleucine (Figure 1B) and a transition of nucleotide 106546 from T to C producing a silent mutation within ORF62/71 (Figure 1C). The nucleotide change in ORF62/71 was also previously shown in the sequencing of an ORF61 deletion virus that was generated from the same parental SVV cosmid system [11-13]. SVV ORF22 is a putative tegument protein based on the function of herpes simplex virus type-1 (HSV-1) UL36 homolog. The missense mutation in ORF22 did not render SVV BAC derived virus noninfectious or hamper replication kinetics and plaque size *in vitro*[11].

### Disease severity and viral load

**Figure 2:**
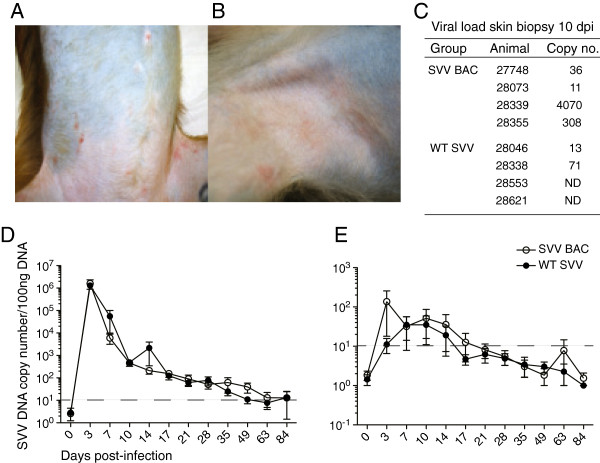
**Varicella and viral load in SVV BAC and WT SVV infected RMs. A** and **B)** Representative examples of varicella in WT SVV and SVV BAC infected RMs. **(A)** SVV BAC infected RM on the trunk region and **(B)** WT SVV infected RM on the axillary region at 7dpi. SVV DNA viral load in **C)** skin biopsy at 10 dpi measured by quantitative PCR using primers and probe specific for SVV ORF21. Average copy number per microgram of DNA. SVV DNA viral load in **D)** BAL and **E)** whole blood was measured by quantitative PCR using primers and probe specific for SVV ORF21 from SVV BAC infected RMs (open circle) and WT SVV infected RMs (closed circle). Dashed line indicates limit of detection.

Rhesus macaques (RMs) were infected with SVV BAC or WT SVV at 4×10^5^ PFU intrabronchially (n=4 per group, sex and age matched). We investigated the pathogenesis of BAC derived SVV *in vivo* by measuring disease progression, viral replication, immune response, and the establishment of latency compared to WT SVV. We collected bronchoalveolar lavage (BAL) cells and blood (peripheral blood mononuclear cells, PBMC) at various days post-infection (dpi) and sensory ganglia were collected at necropsy (84–86 dpi). All infected RMs displayed hallmarks of SVV infection including the development of rash, which lasted between 7 and 10 days. A representative RM infected with SVV BAC at 7 dpi is shown in Figure 2A and a representative RM infected with WT SVV at 7 dpi is shown in Figure 2B. A lesion area was biopsied at 10 dpi and viral loads were measured by quantitative real-time PCR (Figure 2C). By 10 dpi, we were able to detect viral DNA in all RMs except RMs 28553 and 28621 infected with WT SVV. SVV viral loads were also measured by quantitative real-time PCR in BAL cells and whole blood samples. BAL cell viral loads peaked at 3 dpi in both SVV BAC and WT SVV infected RMs then decreased to levels near or below our limit of detection by 63 dpi (Figure 2C). SVV viral loads in whole blood are significantly lower than in BAL cells, though we were able to detect SVV DNA in whole blood between 3 and 14 dpi in RMs infected with SVV BAC or WT SVV and then viral loads decreased to levels near or below our limit of detection (Figure 2D). Therefore, the ability of SVV BAC to replicate *in vivo* was comparable to WT SVV.

### Cytokine and chemokine levels in BAL supernatant and plasma

**Figure 3:**
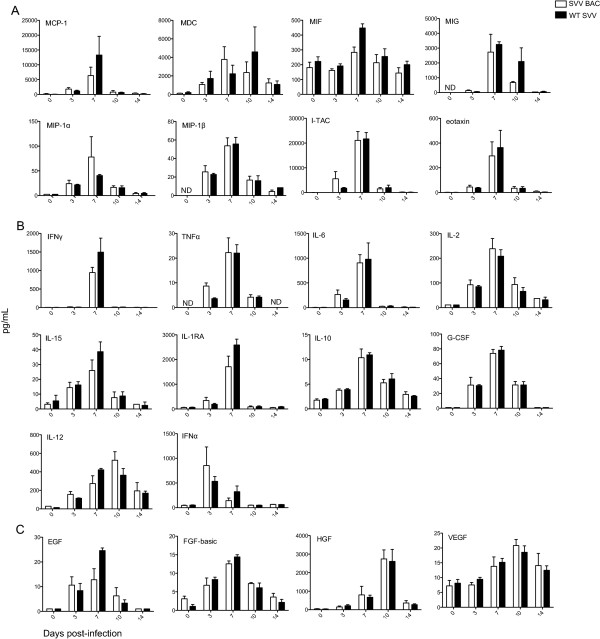
**Chemokine and cytokine response in BAL fluid.** Levels of **(A)** chemokines, **(B)** cytokines and **(C)** growth factors were determined using multiplex technology in BAL supernatants. Concentrations of IFNα were determined by IFNα ELISA. Average pg/mL ± SEM. SVV BAC (white bar) and WT SVV (black bar).

We measured the concentrations of multiple chemokines (Figure 3A), cytokines (Figure 3B), and growth factors (Figure 3C) in BAL fluid and plasma (data not shown) by multiplex technology. In BAL fluid infection with either SVV BAC or WT SVV induced production of several key chemokines, including MCP-1 (recruits monocytes, memory T cells, DCs [14]), MDC (recruits monocytes, monocyte-derived DCs, and NK cells [15]), MIF (inflammatory and atherogenic leukocyte recruitment [16]), MIG (recruits T cells [17]), MIP-1α (recruits and activates polymorphonuclear leukocytes [18]), MIP-1β (recruits NK cells and monocytes [18]), I-TAC (recruits T cells [19]), and eotaxin (recruits eosinophils [20]) (Figure 3). Concentrations of these chemokines peaked at 7 dpi and returned to baseline by 14 dpi.

Concentrations of pro-inflammatory cytokines IFNγ (involved in anti-viral activities and differentiation of T helper 1 (Th1) subsets [21]), TNFα (systemic inflammation [22]), IL-6 (pro- and anti-inflammatory responses [23]), IL-2 (T cell proliferation and homeostasis [24]) and IL-15 (proliferation of T cells and NK cells [25]) peaked at 7 dpi. The concentrations of IL-1 receptor antagonist (RA), which prevents IL-1 from signaling through the IL-1R [26] and IL-10 (pleiotropic activities in inflammation and immune regulation [27]) peaked at 7 dpi. G-CSF, a pleiotropic cytokine, produced by endothelium, macrophages and other immune cells, which stimulates the survival, proliferation, differentiation, and function of neutrophils also peaked at 7 dpi [28]. Levels of IL-12, important in the differentiation of naïve T cells into CD4 Th1 cells [29], peaked at 10 dpi. Lastly, the concentration of IFNα, a type I interferon that is important in anti-viral immunity, peaked at 3 dpi.

Growth factors EGF (stimulates cell growth, proliferation and differentiation [30]), and FGF-basic (multifunctional protein involved in angiogenesis and wound healing [31]) concentrations in BAL fluid peaked at 7 dpi. While levels of growth factors HGF (regulates cell growth, cell motility, and morphogenesis, and acts primarily upon epithelial and endothelial cells [32]) and VEGF (stimulates vasculogenesis and angiogenesis [33]) peaked at 10 dpi.

Concentrations of IL-1β, IL-4, IL-5, IL-8, IL-17, GM-CSF and RANTES (CCL5) and in BAL fluid were below our limit of detection (data not shown). We did not detect any significant differences in concentrations of chemokines, cytokines or growth factors between RMs infected with SVV BAC or WT SVV.

We also measured the concentration of the above chemokines, cytokines, and growth factors in plasma (data not shown). However, we were only able to detect changes in the levels of IFNγ, which peaked at 7 dpi and returned to baseline by 10 dpi and no significant differences were detected between cohorts.

### B cell and antibody response to SVV BAC

**Figure 4:**
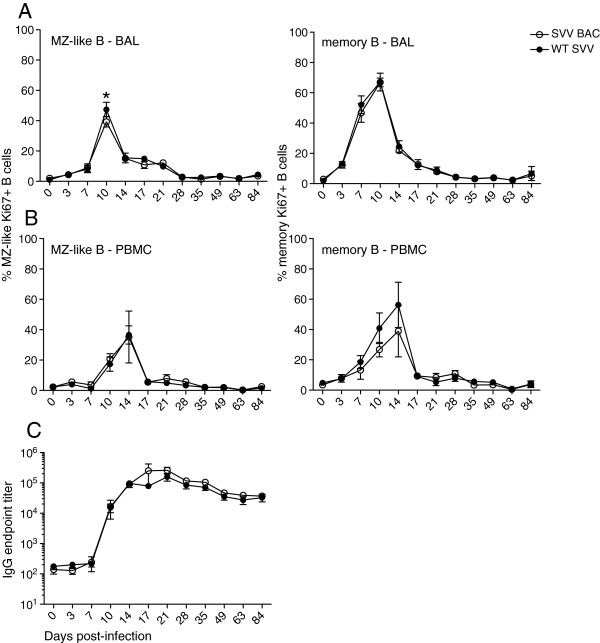
**B cell proliferation and IgG production.** The frequency of proliferating in MZ-like and memory B cell subsets was measured using flow cytometry based on the expression of Ki67 in **(A)** BAL and **(B)** PBMC. SVV-specific **(C)** IgG antibody endpoint titers were measured by standard ELISA. SVV BAC (open circle) and WT SVV (closed circle). Average ± SEM *p<0.05.

We compared the magnitude and kinetics of the B cell response as well as the generation of SVV-specific IgG antibody titers post-infection in RMs infected with SVV BAC or WT SVV. The expansion of B cells is measured based on expression of Ki67, a nuclear protein involved in DNA replication [34] by flow cytometry. SVV infection induces the proliferation of B cells indicated by an increase in the frequency of Ki67 positive cells on days 7 to 14 compared to day 0 in marginal zone (MZ)-like (CD27^+^IgD^+^) and memory (CD27^+^IgD^−^) B cells, in BAL cells (Figure 4A) and PBMC (Figure 4B). In BAL cells and PBMC we measured similar proliferation of both subsets of B cells and no statistical differences in RMs infected with either SVV BAC or WT SVV except for at 10 dpi in BAL cells of WT SVV infected RMs we detected higher proliferation of MZ-like B cells (p<0.05) compared to SVV BAC. We also measured SVV-specific IgG (Figure 4C) antibody endpoint titers in plasma using standard ELISA. The kinetics of IgG production were comparable during SVV BAC and WT SVV infection of RMs, the titers peaked around day 14 post-infection and remained stable until necropsy.

### T cell response to SVV BAC

**Figure 5:**
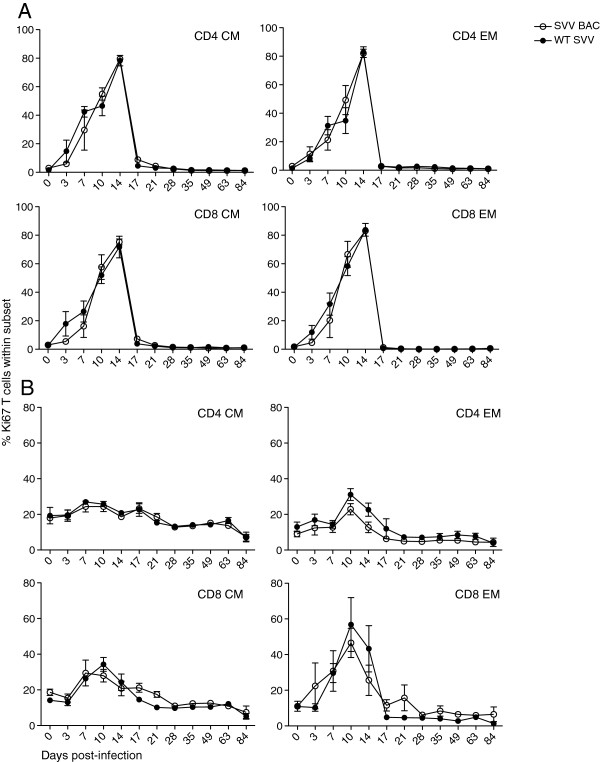
**T cell proliferation.** The frequency of proliferating CD4 and CD8 T cells was measured by flow cytometry based on the expression of Ki67 in central memory (CM) and effector memory (EM) subsets in **(A)** BAL and **(B)** PBMC, average ± SEM. SVV BAC (open circle) and WT SVV (closed circle).

Naïve T cells following antigen encounter become activated, proliferate, and differentiate into central memory (CM, CD28^+^CD95^+^) and effector memory (EM, CD28^−^CD95^+^) T cells. We compared the kinetics and magnitude of the T cell response by measuring the frequency of Ki67 positive CM and EM T cells subsets in BAL cells (Figure 5A) and PBMC (Figure 5B) in SVV BAC or WT SVV infected RMs. SVV BAC and WT SVV infection induced strong T cell proliferation in BAL cells as shown by an increase in Ki67 positive T cells from days 7 to 17 post-infection. Within PBMC, T cell proliferation was detected in CD4 EM, CD8 CM and CD8 EM subsets but the magnitude was significantly reduced compared to the proliferation observed in BAL cells. Similarly though, the magnitude and kinetics was comparable in RMs infected with SVV BAC and WT SVV.

**Figure 6:**
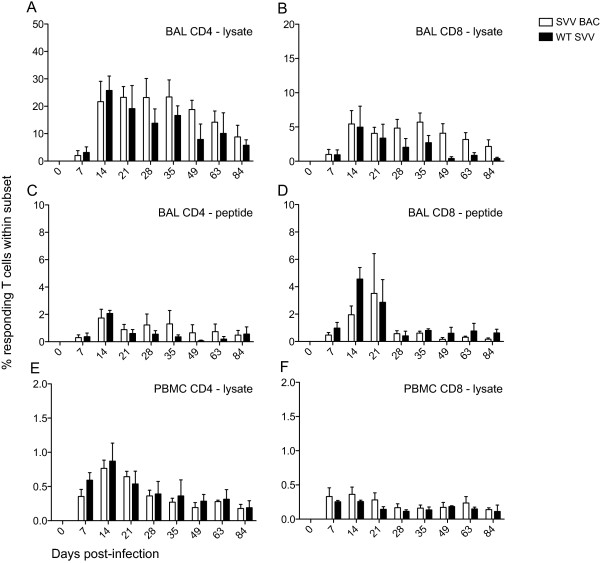
**The frequency of responding SVV-specific T cells.** The frequency of SVV-specific T cells in **(A**-**D)** BAL and **(E** and **F)** PBMC producing IFNγ, TNFα and IFNγ/TNFα was measured by intracellular cytokine staining following stimulation with either **(A**, **B**, **E** and **F)** SVV lysate or **(C** and **D)** overlapping peptide pool (SVV ORFs 4, 31, 61 and 63), average ± SEM. SVV BAC (white bar) and WT SVV (black bar).

Additionally, we determined the frequency of SVV-specific T cells within CD4 and CD8 T cell populations by measuring the combined number of IFNγ-, TNFα- and, IFNγ/TNFα-producing cells following stimulation with either SVV lysate or a SVV overlapping peptide pool covering ORFs 4, 31, 61 and 63 using intracellular cytokine staining (ICS). We stimulated both BAL cells (Figure 6A-D) and PBMC (Figure 6E,F) isolated from infected RMs at different dpi. Within BAL cells of SVV BAC or WT SVV infected RMs, SVV-specific CD4 and CD8 T cells were detected 7 dpi, their frequency peaked between 14 and 21 dpi and declined to a memory set point. There were no statistically significant differences between animals infected with SVV BAC or WT SVV, and both cohorts did not produce a measurable CD4 or CD8 response in PBMCs following stimulation with overlapping viral peptide pools (data not shown).

### SVV viral load in sensory ganglia

SVV DNA viral loads in sensory ganglia were measured by quantitative PCR (Table 1). The viral loads reported in Table 1 reflect SVV genome copy numbers in a portion of the ganglia and are therefore not representative of the entire organ. Four of the RMs went on to further studies therefore Table 1 shows the latent viral loads for two animals from each cohort. We detected SVV DNA within at least one sensory ganglia of each RM infected with SVV BAC and WT SVV indicating that the SVV BAC, like WT SVV is able to traffic to the sensory ganglia, the site of SVV latency.

**Table 1:** SVV viral load in sensory ganglia

**SVV BAC animal ID**	**Sample**	**Copy no.**^**a**^	**WT SVV animal ID**	**Sample**	**Copy no.**^**a**^
28339	TG	576	28046	TG	14
	DRG-C	ND		DRG-C	ND
	DRG-T	1526		DRG-T	ND
	DRG-L/S	67		DRG-L/S	ND
28355	TG	ND	28553	TG	41
	DRG-C	17		DRG-C	ND
	DRG-T	ND		DRG-T	ND
	DRG-L/S	ND		DRG-L/S	ND

^a^average copy number per ug of DNA.

DRG-C, cervical dorsal root ganglia; DRG-T, thoracic dorsal root ganglia; DRG-L/S, lumbar/sacral dorsal root ganglia; *ND*, not detected.

## Discussion

Cloning viral genomes as bacterial artificial chromosomes (BAC) is an efficient tool to manipulate the viral genome facilitating the study of viral genes *in vitro* and *in vivo*. BACs were constructed for the VZV parental Oka virus and the vaccine Oka virus [35-38]. All VZV ORFs have been deleted using BACs or cosmids and tested in cell culture providing valuable information on which VZV genes are essential for viral replication *in vitro* (reviewed in [39,40]). Subsequent *in vivo* analysis of VZV mutant viruses using the SCID-hu mouse model showed that VZV ORFs 1, 2, and 3 are dispensable for viral replication [38], whereas VZV ORF23, which was dispensable for replication in culture, was found to be required for replication in human skin xenografts [41]. Similarly, VZV deleted for ORF7 replicated in MeWo cells but *in vivo* ORF7 was shown to be important for neuroinvasion [40,42]. Lastly, mutating the furin recognition site of VZV ORF31 (gB), an essential viral protein, did not affect VZV replication *in vitro* but attenuated viral replication *in vivo*[43]. Studying VZV mutant viruses in the SCID-hu mouse model has provided valuable insight into VZV biology, further evaluation of the role of different VZV ORFs in pathogenesis and the immune response *in vivo* is hampered by the fact that VZV is an obligate human pathogen. SVV is a simian homolog of VZV that causes varicella and zoster in nonhuman primates [4-6].

Recently, the SVV genome was cloned as a BAC and SVV virus generated using the SVV BAC genetic system was found to be similar to WT SVV *in vitro*[11]. Mutagenesis of SVV ORF10 showed that SVV ORF10 is nonessential for replication *in vitro*[11]. Additionally, generation of a SVV ORF 63/70 mutant demonstrated impaired growth in Vero cells [44]. In the present study we further the analysis of SVV BAC by infecting rhesus macaques and investigating the pathogenesis of SVV BAC compared to WT SVV *in vivo*. The combination of the rhesus macaque animal model and the SVV BAC will provide a robust tool to examine viral ORFs important for pathogenesis and help to target VZV ORFs that will improve vaccine efficacy.

To compare the genome of the SVV BAC to WT SVV we utilized comparative genomic hybridization. CGH analysis was employed as a cost-effective and accurate strategy to analyze genomic DNA from multiple viruses. This technique is sensitive enough to detect single base changes in addition to insertions, deletion or rearrangements in the genome [45-47]. We sequenced two sites in the SVV BAC genome that displayed variations in hybridization when compared to WT SVV. In ORF22 a point mutation at nucleotide 41990 was found producing an amino acid change from valine to isoleucine. SVV and VZV ORF22 are putative tegument proteins based on homology to HSV-1 UL36 [48]. UL36 is a HSV-1 late gene and the HSV-1 virion contains 100–150 copies of UL36 [49,50]. UL36 is essential to HSV-1 replication and the phenotype of a null mutant virus showed accumulation of capsids containing cytosolic DNA that did not mature into enveloped virions [51-53]. Though, the evidence suggests that the amino acid change in SVV BAC does not constitute a significant change in the protein. *In vitro* SVV BAC displays similar plaque size and replication kinetics in Vero cell monolayers as compared to WT SVV [11]. Our data *in vivo* shows that SVV BAC displays similar replication kinetics, immune response and establishment of latency compared to WT SVV. Potentially the position of the amino acid change or that the change is a nonpolar side chain to a nonpolar side change allows for WT behavior.

The replication kinetics of SVV BAC in the bronchoalveolar lavage cells (the site of virus inoculation) and in the peripheral blood was statistically similar to WT SVV. Related, the spread of varicella rash was also similar between cohorts and lasted between 7 and 10 days post-infection. In Figure 2A and B we show pictures of two RMs infected with SVV BAC or WT SVV, which showed representative rash and also illustrates the variation in rash spread we see in our animals. In two animals infected with WT SVV, we did not detect viral DNA in the skin lesions but due to the nature of the skin punch biopsies as well as the timing of the rash, which varies between animals usually between 7 to 10 days post-infection, we may not have obtained a lesion spot with detectable viral DNA.

We also followed the immune response to SVV BAC during acute infection and found SVV BAC to elicit a parallel immune response *in vivo*. We analyzed the proliferation kinetics of antigen experienced B cells, the production of SVV-specific IgG antibodies as well as the proliferation kinetics of memory T cells and the IFNγ/TNFα response of SVV-specific CD4 and CD8 T cells, and each parameter was analogous. We did measure a statistical difference in MZ-like B cells at 10 dpi in the BAL where RMs infected with WT SVV displayed a higher peak percentage of Ki67 positive cells. However, this difference did not translate to a higher antibody titer in the WT SVV infected RMs. This difference could be due to the small sample size (n=4) and the outbred nature of rhesus macaques.

Infection with SVV BAC also resulted in a comparable upregulation of chemokines, cytokines, and growth factors during the early stages of acute infection in the lungs compared to RMs infected with WT SVV. Peak levels of IFNα, an important antiviral cytokine, occurred at 3 dpi, which corresponds to peak viral loads in the lungs also at 3 dpi. Type I interferons are early immune effectors and are important cytokines during initial infection to limit viral replication and spread, including herpesviruses [54-56]. The concentrations of several T cell-recruiting chemokines (MCP-1, MIG, I-TAC) peaked 7 days prior to the observed peak in proliferating T cells in BAL samples. We also detected increased concentrations of TNFα (3 dpi) and IFNγ (7 dpi), which is indicative of Th1 immune responses and correlates with an increase in CD4 CM in BAL samples. Peak concentrations of IL-2 were detected 7 days prior (7 dpi) to the peak proliferation of CD4 and CD8 T cells (14 dpi). Levels of IL-12, which play a role in enhancing cytotoxic function of CD8 T cells, peaked 4 days before peak proliferation of CD8 T cells in BAL samples. Interestingly, we also detected an increase in growth factors in the BAL fluid, which peaked from 7 to 10 days post-infection. Many of these growth factors are involved in wound healing and might represent a response to tissue injury induced by SVV replication within the lungs (site of inoculation).

Lastly we found that both SVV BAC and WT SVV established latency within the sensory ganglia. Viral DNA was detected in at least one ganglion from each RM measured by quantitative PCR. In summary, SVV BAC is as pathogenic *in vivo* as WT SVV. Future studies will utilize the SVV BAC genetic system to generate knockout viruses to help characterize the role of SVV genes in acute infection, the establishment and maintenance of latency, and reactivation *in vivo*, a critical step in the understanding of viral factors that impact VZV pathogenesis and the immune response to VZV.

## Materials and methods

### Cells and viruses

Bacterial artificial chromosome (BAC)-derived SVV was generated from self-excisable pSVV-BAC resulting in complete excision of plasmid sequences from the virus genome [11]. Wild-type (WT) simian varicella virus (SVV, *Cercopithecine herpesvirus* 9) and SVV BAC were propagated as previously described, briefly Vero cells maintained in Eagle’s minimal essential medium (EMEM) supplemented with 5% newborn bovine serum, penicillin and streptomycin, WT SVV and SVV BAC infected Veros were harvested by scraping and frozen in Vero media supplemented with 10% dimethyl sulfoxide (DMSO) [11]. Virus stocks were titered by plaque assay on primary rhesus fibroblasts maintained in Dulbecco’s modified Eagle’s Medium (DMEM) supplemented with 10% fetal bovine serum and penicillin, streptomycin and L-glutamine. WT SVV cell lysate was obtained by scraping infected primary rhesus fibroblasts at the height of CPE followed by centrifugation and sonication using 7 pulses of 70–80 Watts (Sonicator 3000, Misonix Inc., Farmingdale NY) and frozen at −80°C.

### Animals and sample collection

All rhesus macaques were housed at the Oregon National Primate Research Center and were handled in accordance with good animal practices as defined by the Office of Laboratory Animal Welfare. Animal work was approved by the Oregon National Primate Research Center Institutional Animal Care and Use Committee. Rhesus macaques (RM, *Macaca mulatta*) were SVV seronegative prior to infection measured by ELISA. RMs (n=4 per group) were infected intrabronchially with 4×10^5^ PFU WT SVV or SVV BAC infected Veros. Peripheral blood mononuclear cells (PBMC) and bronchoalveolar lavage (BAL) cells were collected from rhesus macaques as previously described [5]. Animals were euthanized at 84 to 86 days post-infection. Sensory ganglia: trigeminal ganglia (TG), cervical, thoracic and lumbar-sacral dorsal root ganglia (DRG-C, DRG-T, and DRG-L/S respectively) were divided, flash frozen and stored at −80°C until analysis.

### Comparative genome analysis of SVV BAC and SVV WT DNA

A microarray hybridization-based method was used to compare SVVΔORF61 genomic DNA (test) to WT SVV (reference) DNA provided by NimbleGen Systems, Inc. (CGS 385K Mutation Mapping array Phase 1, Madison WI). Design of the microarray used published sequence data for the Delta herpesvirus strain of SVV (NC_002686, [8]). The oligonucleotides were 29–39 bp in length and tiled throughout the genome every 7–8 bases on both forward and reverse strands. Viral DNA was isolated from nucleocapsids purified from SVV-infected Vero cells as previously described [7]. Hybridization data was analyzed using SignalMap software (NimbleGen Systems, Inc., Madison WI). The identified mismatches were directly sequenced from PCR products obtained from amplifying the surrounding sequence. The primers employed for Figure 1 include: (B) primer 1, 5′-CCATATGTACCAACGGGAACA-3′ and primer 2, 5′-AAGCATGCATTTTCGATTGGA-3′; (C) primer 1, 5′-GCCTGGAGCCCAGATATTCGA-3′ and primer 2, 5′-ACGGTGTGCGTGGATGCATCA-3′.

### DNA extraction and quantitative real-time PCR (qPCR)

DNA was extracted from heparinized whole blood (WB), BAL cells, and portions of frozen ganglia using ArchivePure DNA Cell/Tissue Kit (5 PRIME, Gaithersburg MD) according to the manufacturer’s protocol. SVV DNA viral loads in WB, BAL cells and sensory ganglia were measured by qPCR using Maxima Probe/ROX qPCR Master Mix (2X) (Fermentas, Glen Burnie MD) and primers/Taqman probe specific for SVV ORF21. Following an initial 10 minute 95°C step, 40 cycles of 15 sec at 95°C and 1 minute at 60°C were completed using StepOnePlus (Life Technologies, Carlsbad CA). SVV BAC DNA was used as quantification standards [11].

### Cytokine analysis

Plasma and BAL supernatant samples (stored at −80°C) were thawed and analyzed using Cytokine Monkey Magnetic 28-plex panel as per the manufacturer’s instructions (Life Technologies). IFNα levels were measured using Cynomolgus/Rhesus IFNα Serum ELISA Kit according to the manufacturer’s instructions (PBL Interferon Source, Piscataway NJ). Samples were run in duplicate. Values below the limit of detection were designated as ND, or not detected.

### Enzyme-linked immunosorbent assay (ELISA)

ELISA plates were coated with SVV lysate overnight at 4°C, blocked with 5% milk in wash buffer (0.05% Tween in PBS) for 1 h at room-temperature (RT), washed three times with wash buffer, and incubated with heat-inactivated (55°C, 30 min) plasma samples in 3-fold dilutions in duplicate for 1 h. After washing three times with wash buffer, horseradish peroxidase (HRP)-conjugated anti-rhesus IgG (Nordic Immunology, Netherlands) was added for 1 h, followed by addition of chromagen *o*-phenylenediamine•2HCl (OPD) (Sigma, St Louis MO) substrate for 20 minutes to allow detection and quantitation of bound antibody molecules. The reaction was stopped with the addition of 1 M HCl. The optical density was measured at 490 nm using an ELISA plate reader (SpectraMax 190, Molecular Devices, Sunnyvale CA). Endpoint IgG titers were calculated using log-log transformation of the linear portion of the curve with 0.1 optical density (OD) units as the cut-off. Titers were standardized using a positive control sample included with each assay.

### Measurement of T cell and B cell frequency and proliferation

BAL cells and PBMC were surface stained with antibodies against 1) CD4 (eBioscience, San Diego CA), CD8β (Beckman Coulter), CD28, and CD95 (BioLegend, San Diego CA) to delineate the naive (CD28^+^CD95^−^), central memory (CD28^+^CD95^+^), and effector memory (CD28^−^CD95^+^) T cell subsets; 2) CD20 (Beckman Coulter, Brea CA), IgD (Southern Biotech, Birmingham AL), and CD27 (BioLegend) to delineate naïve (CD20^+^IgD^+^CD27^−^), marginal zone-like (MZ-like) (CD20^+^IgD^+^CD27^+^), and memory (CD20^+^IgD^−^CD27^+^) B cell subsets. Cells were fixed and permeabilized according to manufacturer recommendations (BioLegend) before the addition of Ki67-specific antibody (BD Biosciences, San Jose, CA). The samples were analyzed using the LSRII instrument (Beckton, Dickinson and Company, San Jose CA) and FlowJo software (TreeStar, Ashland OR).

### Intracellular cytokine staining

BAL cells and PBMC were stimulated with SVV lysate (1 μg) or SVV overlapping peptide pool containing open reading frames (ORFs) 4, 31, 61 and 63 for 1 h followed by addition of Brefeldin A (Sigma, St Louis MO) to block cytokine export for 14 h. After stimulation cells were surface stained with antibodies against CD4, and CD8β, as described above. Samples were fixed, permeabilized (BioLegend) and dual-stained using antibodies against IFNγ (eBioscience) and TNFα (eBioscience). Samples were analyzed using the LSRII instrument and FlowJo software.

### Statistical analysis

Statistical analysis and graphing was conducted with GraphPad Prism software (GraphPad Software Inc., La Jolla CA). Significance values for Figures 2, 3, 4, 5, and 6 utilized repeated measures of ANOVA with the Bonferroni post-test to explore differences between groups (SVV BAC and WT SVV) at each time-point.

## Competing interests

The authors declared that they have no competing interests.

## Authors’ contributions

Study design: IM and CM; data collection: CM, JD, KH, FE, NA; data interpretation and manuscript preparation: IM and CM. WG provided SVV BAC and WT SVV. All authors read and approved the final manuscript.
